# Hand Grip Strength Vs. Sprint Effectiveness in Amputee Soccer Players

**DOI:** 10.1515/hukin-2015-0099

**Published:** 2015-01-12

**Authors:** Marta Wieczorek, Wojciech Wiliński, Artur Struzik, Andrzej Rokita

**Affiliations:** 1 Department of Special Physical Education Didactics, The University School of Physical Education in Wrocław, Poland; 2 Department of Team Sport Games The University School of Physical Education in Wrocław, Poland

**Keywords:** amputee athletes, running velocity, soccer

## Abstract

Amputee soccer is one of the types of soccer designed for the disabled, especially those who have undergone amputations, as well as those with extremity dysfunction. The objective of the study was to find the relationship between hand grip strength and sprint time in amputee soccer players. Thirteen field amputee soccer players participated in the study. A SAEHAN hydraulic hand dynamometer manufactured by Jamar was used for hand grip strength measurements. The sprint running test was conducted over a distance of 30 m. The Fusion Smart Speed System was employed for running time measurements. No statistically significant relationships were found between hand grip strength of the left or right hand, and sprint times over 1, 5, 10, 15, 20, 25 and 30 m. Analysis of the running velocity curve of the subjects showed an interesting profile characterized by a 15 meter-long acceleration phase and a significant velocity increase over a distance of 20 – 25 m. The study suggests that there is no relationship between hand grip strength and sprint effectiveness in amputee soccer players. The specificity of locomotion with the use of elbow crutches among elite Polish amputee soccer players probably accounts for the profile of the sprint velocity curve. Extension of the acceleration phase in the sprint run and a velocity increase in the subsequent part of the run were observed.

## Introduction

Amputee soccer is one of the types of soccer designed for the disabled, especially those who have undergone amputations as well as those with extremity dysfunction. In amputee soccer, outfield players have unilateral amputations of lower extremities (above the ankle) and move on the pitch with the use of elbow crutches and kick the ball only with their remaining foot. Goalkeepers, however, have a unilaterally amputated upper extremity (above the wrist) and are not permitted to leave the penalty area. In amputee soccer, also, people with congenital motor disorders representing a similar level of functional capacity may compete as amputee soccer players. Other examples of adapting amputee soccer rules consist in reducing dimensions of the pitch and goal, decreasing the number of players and duration of play. These modifications make amputee soccer worth consideration for its unique specificity when conducting research.

As indicated in the to-date scientific observations, amputee soccer demands a considerable level of core stability, balance and trunk strength from its players (Yazicioglu et al., 2007; Aytar et al., 2012). At the same time, it is important to remember that locomotion with the use of elbow crutches may cause pathologic changes in the wrist joints (Requejo et al., 2005), biceps brachii tendon (Krüger and Bischof-Léger, 2008), as well as haematomas and pain through the pressure load on the intermediate and ulnar quadrants of the forearm (Fischer et al., 2014). On the other hand, Ramazanoğlu et al. (2013) paid attention to more favorable gait parameters obtained during locomotion by means of elbow crutches than prostheses. A crutch-assisted gait, additionally, supported a symmetric locomotion pattern in amputee soccer players (Ramazanoğlu et al., 2013). Haubert et al. (2006) found that elbow crutches enabled the achievement of enhanced stride maneuverability and length as compared to other assistive gait devices, but may also cause shoulder joint strain.

In soccer, however, it is not the gait but sprint running along a straight line that is one of the most important forms of activities among players assisting in goal-scoring as well as goal-scorers themselves. Analysis of video recordings of 360 goals from the second half of the 2007/2008 season of the first German national league showed that 45% of the goals were scored following sprinting in a straight line by players (Faude et al., 2012). Ferro et al. (2014) pointed to the significance of sprint velocity, particularly over a distance of 0–10 m, which was related with the player’s position on the field. Among professional players, the highest running velocity is achieved by forwards, then by central midfielders, wide midfielders, defenders and finally by central backs (Ferro et al., 2014). According to Simim et al. (2013), the position of amputee soccer players on the field (defender, midfielder, forward) does not differentiate them in terms of running velocity over a distance of 20 m, agility and endurance, as well as anthropometric parameters such as: body height, body mass or BMI. The only exception was the percentage of fat content (BF%), that was lower in midfielders than in defenders (Simim et al., 2013). Özkan et al. (2012) did not report any statistically significant relationships between body composition and sprint times over distances of 10, 20 and 30 m in amputee soccer players. The sprint time at 10, 20 and 30 m showed, however, negative relationships with power generated during the countermovement jump (CMJ) and the squat jump (SJ). The authors also indicated that body somatotype of amputee soccer players played an important role in jumping and sprint performance (Özkan et al., 2012).

Hand grip strength may attain higher values as a result of proper training (Fidelus et al., 1992; Fallahi and Jadidian, 2011). In young judokas (males), hand grip strength achieves greater values in higher weight categories, whereas in women it enables the prediction of results of competitions (Sánchez et al., 2011). Due to the use of elbow crutches by amputee soccer players, hand grip strength improves on an ongoing basis. In view of the presented data, it seems necessary to study the relationship between locomotion effectiveness and hand grip strength.

Therefore, the authors decided to investigate the relationship between hand grip strength and sprint time (locomotion effectiveness) in amputee soccer players, that had not yet been identified. Thus, the following research questions were formulated:

1. Is there a relationship between hand grip strength and sprint time over a distance of 30 m in elite amputee soccer players?
2. What is the profile of the sprint velocity curve among elite Polish amputee soccer players and how does it compare to fully-abled players?

## Material and Methods

### Participants

Thirteen representatives of Poland in amputee soccer participated in the study. Goalkeepers, due to their different anthropometric characteristics were excluded from the analyses. Before the research began, the volunteers signed an informed consent form. The subjects filled in the survey questionnaire, in which they were asked to provide information concerning specific nature of their disability, training experience and the level of sports activity. The lower extremity amputation level among the subjects was as follows: the knee joint or above (4 subjects), below the knee joint but above the ankle (7 subjects), anomalies connected with the structure of the thigh bone (2 subjects). Besides training in amputee soccer two participants used exclusively elbow crutches, five of them used only prostheses, whereas six players used both means for locomotion simultaneously. The anthropometrical and training characteristics of the amputee players (*n* = 13) are presented in Table 1.

### Procedures

A SAEHAN hydraulic hand dynamometer manufactured by Jamar was used for left and right hand grip strength measurements. The subjects performed the test while sitting with arms along the body, with the elbow joint in 90° flexion and the forearm and the wrist in a neutral position, which corresponded to the standard measurement procedure (Reddon et al., 1985). Players gripped the handle of the dynamometer twice with the right and left hand, and for further analysis only higher values of grip strength for each subject were used (for both extremities).

Every player performed a 30 m run along a straight line twice. For the purposes of analysis, only the trial with a shorter 30 m running time by each of the subjects was taken into account. With regard to the running times in particular sections, a Fusion Smart Speed System (Fusion Sport, Coopers Plains, QLD, Australia) was applied. It consists of gates constituted by photocells containing heads emitting infrared light and mirrors reflecting the light. The 30 m straight line running trial was carried out with the use of 8 gates. A distance of 3 m was maintained between the photocells and mirrors. Beginning with the start line, the next gates were placed onwards which recorded the time when IR beams were broken. The gate spaced 30 m away from the start line marked the finish line. During runs splits were also recorded at 1, 5, 10, 15, 20 and 25 m. In this manner, mean running velocity was calculated in individual sections. The subjects began running from a standing start and they decided themselves when to start running. Crutches were held stretched out by subjects and did not cross the starting line.

### Statistical Analysis

The obtained data underwent statistical analysis for the purpose of observation of co-occurrence of variables (left and right hand grip strength vs. sprint time over distances of: 0–1, 0–5, 0–10, 0–15, 0–20, 0–25 and 0–30 m) using the *r*-Pearson correlation coefficient (near normal distribution of variables). Significance of differences between mean running velocities in individual sections was investigated using a *t*-Student test for dependent variables.

## Results

Mean value (±SD) of the right hand grip strength among the subjects was 45.9 ± 12.6 kG, while in the left hand 45.6 ± 10.4 kG. Mean sprint times among amputee players were as follows in respective sections: 0–1 m: 0.33 ± 0.06 s; 0–5 m: 1.16 ± 0.09 s; 0–10 m: 2.08 ± 0.11 s; 0–15 m: 2.93 ± 0.14 s; 0–20 m: 3.80 ± 0.19 s; 0–25 m: 4.62 ± 0.24 s and 0–30 m: 5.47 ± 0.29 s. No statistically significant correlations (r-Pearson) were found between left and right hand grip strength, and sprint times in particular sections (0–1, 0–5, 0–10, 0–15, 0–20, 0–25 and 0–30 m). The obtained values of correlation coefficients are presented in Table 2.

**Figure 1:**
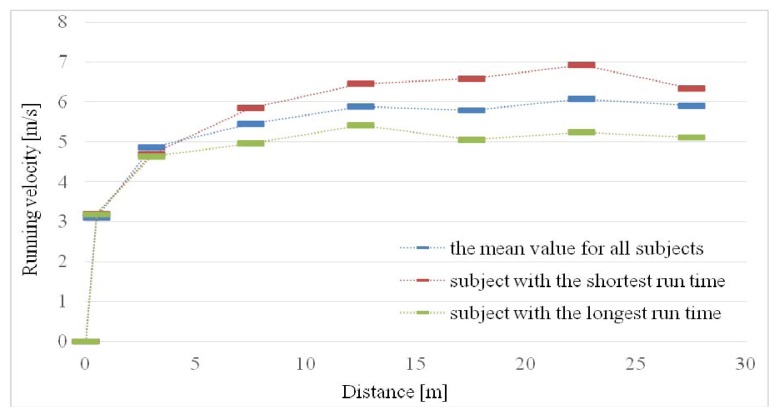
Velocity curves in sprint running for Polish amputee soccer players. Points on the graph signify mean velocity achieved by players in sections: 0–1, 1–5, 5–10, 10–15, 15–20, 20–25 and 25–30 m.

Table 3 contains mean velocity in specific sections of the sprint over a distance of 30 m. Statistically significant differences were reported in mean running velocities between sections: 0–1 vs. 1–5 m (increase), 1–5 vs. 5–10 m (increase), 5–10 vs. 10–15 m (increase), 15–20 vs. 20–25 m (increase) and 20–25 vs. 25–30 m (decrease). The difference between sections 10–15 and 15–20 m was not statistically significant. Running velocity curves over a distance of 30 m in elite Polish amputee soccer players (mean value for all subjects and players with the shortest and longest running times, respectively) are presented in Figure 1.

## Discussion

The obtained results suggest that hand grip strength has no relationship with sprint effectiveness in elite amputee soccer players. The expected high values of hand grip strength among the subjects as permanent users of elbow crutches were only slightly higher than the results achieved by wheelchair basketball players from category A (1.0–2.5 point): 40.71 ± 9.95, and lower than those achieved by category B players (3.0–4.5 point): 48.29 ± 12.06 (Yanci et al., 2015). Thus, it is possible that short and local isometric effort measured with a hand dynamometer does not correspond to the specific nature of straight line sprint running using elbow crutches, where rather a series of grips determined by hand and forearm muscle endurance occurs. Rokowski and Żak (2010) came to similar conclusions having analyzed the results of professional climbers. The best of them represented the lowest level of hand grip strength measured with a hand dynamometer, but their results were definitely better in a hang trial with a maximum weight on a 2.5 cm bar using a climbing handhold.

It should be noted that the maximum level of absolute and relative hand grip strength may increase with age (Gerodimos, 2012), however, in this respect the group of amputee soccer players undergoing trials was not numerous, but diverse (Table 1). In future studies, it appears reasonable to use anthropometric parameters of the hand (e.g. length of fingers) influencing grip strength (Visnapuu and Jürimäe, 2007; Cortell-Tormo et al., 2013) that might more precisely reflect the specific nature of elbow crutch grip and, at the same time, reveal the possible relationships with straight line sprint time. Little and Williams (2005) suggested that the abilities to accelerate (10 m sprint), maintain maximum velocity (20 m sprint) and agility (Zigzag test) in soccer players should be controlled independently from one another, as they are different components of speed abilities. It may well be that only sprint time with changes of movement direction in amputee soccer players might indicate the relationship with hand grip strength.

Sprint times achieved by Polish elite amputee soccer players over distances of 10, 20 and 30 m were close to those obtained by amputee players from the Karagücü Sports Club, namely: 2.07 ± 0.24, 3.71 ± 0.48 and 5.44 ± 0.77 (Özkan et al., 2013). The specificity of locomotion by means of elbow crutches among Polish elite amputee soccer players probably accounts for the profile of the sprint velocity curve over a distance of 30 m in a straight line (Figure 1). Extension of the sprint acceleration phase (up to 15 m), velocity stabilization (15–20 m) and another (untypical) velocity increase in the subsequent part of the run (20–25 m) (Struzik et al., in press) were noticed (as compared to able-bodied athletes). It is possible that compensating the performance of the run for the use of the upper extremities, relatively stiff elbow crutches and the remaining healthy foot by amputee soccer players is insufficient in order to achieve permanent acceleration lasting until the moment of achieving maximum running velocity. The subsequent running velocity increase (understood as a product of stride length and stride frequency) in the swing-through gait may require, however, a certain correction of the movement pattern, which may enable extension or an increase in frequency of the running stride. The level of elastic properties of the material from which elbow crutches were made is also significant (Hobara et al., 2015).

Thys et al. (1996) reported that optimal gait velocity with elbow crutches was 3.5 km/h, and energy expenditure in the swing-through gait increased with movement velocity 2–3 times higher than in normal walking. Thus, it may be presumed that energy expenditure resulting from alternating work of the upper extremity antagonistic muscle tension (that is supposed to stabilize the body during straight line running) may account for the profile of the velocity curve in sprint running in amputee soccer players. Disabled sprinters, using modern prostheses of the ankle, may achieve maximum running velocity with definitely lower metabolic costs than in the healthy ankle joint complex (Brüggemann et al., 2008). Due to the regulations concerning the rules of amputee soccer, representatives of this discipline may not allow themselves to adopt such solution.

Hobara et al. (2015) reported that the amputee sprinters’ running stride was shorter in comparison to able-bodied sprinters. No significant differences were observed in stride frequency. A shorter stride may result from lower force generation in running-specific prostheses compared with the healthy lower limb (Hobara et al., 2015). The above analogy may also occur while running with elbow crutches. The stance phase, with the use of elbow crutches, may generate insufficient push-off force enabling the lengthening of the running stride, which consequently may translate into the shape of the running velocity curve. Cotte and Chatard (2011) claimed, on the basis of the study on soccer players from the English Premier League, that achieving good results in sprint running over a distance of 30 m was a difficult task and required a high level of lower limb muscle strength.

## Conclusions

1. No statistically significant relationships between right or left hand grip strength and sprint time over a distance of 30 m in a straight line were observed in amputee players.
2. The sprint velocity curve over a distance of 30 m in a straight line in amputee soccer players appeared to be varied as compared to healthy athletes. The sprinting commenced with a 15 m long acceleration phase, followed by relative stabilization of velocity between 15 and 20 m and another increase in running velocity between 20 and 25 m.

## Figures and Tables

**Table 1:** Anthropometric and training characteristics of Polish national men’s amputee soccer players (mean ± SD)

Group (n)	Body height (cm)	Body mass (kg)	Age (years)	Training experience (months)	Sports activity (hours per week)
13	175.4 ± 7.6	70.5 ± 14.9	26.1 ± 7.7	30.8 ± 14.3	6.2 ± 2.3

**Table 2:** Correlation coefficients (r) and probability of type I error (p) between left (SL) and right (SR) hand grip strength and sprint times for sections: 0–1, 0–5, 0–10, 0–15, 0–20, 0–25 and 0–30 m

		Sprint time						

		1 m	5 m	10 m	15 m	20 m	25 m	30 m
SR	*r*	−0.34	−0.49	−0.51	−0.46	−0.35	−0.26	−0.20
	*p*	0.249	0.091	0.077	0.116	0.248	0.400	0.502
SL	*r*	−0.11	−0.26	−0.24	−0.21	−0.13	−0.06	−0.02
	*p*	0.717	0.386	0.433	0.487	0.677	0.855	0.951

**Table 3:** Mean values (±SD) of mean sprint velocity (v) and differences in mean velocity between consecutive sprint sections (Δv) in amputee soccer players

Section:	*v* (m/s)	*Δv* (m/s)	*p*
0 – 1 m	3.1 ± 0.5	1.8 ± 0.5*	0
1 – 5 m	4.9 ± 0.3		
1 – 5 m	4.9 ± 0.3	0.6 ± 0.3*	< 0.00001
5 – 10 m	5.4 ± 0.2		
5 – 10 m	5.4 ± 0.2	0.4 ± 0.2*	< 0.0001
10 – 15 m	5.9 ± 0.3		
10 – 15 m	5.9 ± 0.3	−0.1 ± 0.2	> 0.05
15 – 20 m	5.8 ± 0.4		
15 – 20 m	5.8 ± 0.4	0.3 ± 0.1*	< 0.00001
20 – 25 m	6.1 ± 0.5		
20 – 25 m	6.1 ± 0.5	−0.2 ± 0.3*	< 0.05
25 – 30 m	5.9 ± 0.4		

p – probability of type I error,

* - statistically significant difference for p < 0.05
